# Long Noncoding RNAs as Emerging Regulators of COVID-19

**DOI:** 10.3389/fimmu.2021.700184

**Published:** 2021-08-02

**Authors:** Qinzhi Yang, Fang Lin, Yanan Wang, Min Zeng, Mao Luo

**Affiliations:** ^1^Collaborative Innovation Center for Prevention and Treatment of Cardiovascular Disease of Sichuan Province, Drug Discovery Research Center, Southwest Medical University, Luzhou, China; ^2^Laboratory for Cardiovascular Pharmacology of Department of Pharmacology, The School of Pharmacy, Southwest Medical University, Luzhou, China; ^3^Department of Pharmacy, The Affiliated Hospital of Southwest Medical University, Luzhou, China

**Keywords:** SARS-CoV-2, lncRNAs, virus infections, immune responses, COVID-19

## Abstract

Coronavirus disease 2019 (COVID-19), which has high incidence rates with rapid rate of transmission, is a pandemic that spread across the world, resulting in more than 3,000,000 deaths globally. Currently, several drugs have been used for the clinical treatment of COVID-19, such as antivirals (radecivir, baritinib), monoclonal antibodies (tocilizumab), and glucocorticoids (dexamethasone). Accumulating evidence indicates that long noncoding RNAs (lncRNAs) are essential regulators of virus infections and antiviral immune responses including biological processes that are involved in the regulation of COVID-19 and subsequent disease states. Upon viral infections, cellular lncRNAs directly regulate viral genes and influence viral replication and pathology through virus-mediated changes in the host transcriptome. Additionally, several host lncRNAs could help the occurrence of viral immune escape by inhibiting type I interferons (IFN-1), while others could up-regulate IFN-1 production to play an antiviral role. Consequently, understanding the expression and function of lncRNAs during severe acute respiratory syndrome coronavirus-2 (SARS-CoV-2) infection will provide insights into the development of lncRNA-based methods. In this review, we summarized the current findings of lncRNAs in the regulation of the strong inflammatory response, immune dysfunction and thrombosis induced by SARS-CoV-2 infection, discussed the underlying mechanisms, and highlighted the therapeutic challenges of COVID-19 treatment and its future research directions.

## Introduction

Since December 2019, a respiratory disease caused by severe acute respiratory syndrome coronavirus 2 (SARS-CoV-2) that causes coronavirus illness or coronavirus disease 2019 (COVID-19), has spread to numerous countries worldwide (1–4). As of July 5th, 2021, globally, nearly 184 million people have been diagnosed with COVID-19, and more than 4 million people have died. In the face of the increasingly severe global COVID-19 pandemic, several drugs are already in clinical use, such as antivirals (radecivir, baritinib), monoclonal antibodies (tocilizumab), and glucocorticoids (dexamethasone). Vaccination is the best prophylaxis for the prevention of COVID-19, however, the efficacy of vaccines and onset of adverse reactions vary among individuals. Therefore, it is desperately important to develop safe and effective drugs and vaccines now and in the future.

Increasing studies have reported that long noncoding RNAs (lncRNAs) are involved in various biological regulatory processes, such as inflammation, cell function and immune disorders, and play important roles in the pathogenesis of various diseases, including viral infections and disease progression (5–7). In patients with SARS-CoV-2, several lncRNAs play major regulatory roles in the process of virus infection. For example, lncRNA metastasis-associated lung adenocarcinoma transcript 1 (MALAT1) and nuclear paraspeckle assembly transcript 1 (NEAT1) have been shown to be highly associated with the immune responses and that possibly involved in the inflammatory progression of SARS-CoV-2 infected cells (8, 9). Furthermore, MALAT1 alleviated deep vein thrombosis (DVT) by inhibiting the proliferation and migration of endothelial progenitor cells (EPCs) and involved in thrombosis dissolution *via* regulating the Wnt/β-catenin signaling pathway (10). Collectively, lncRNAs are emerging as key regulators of intense inflammatory response and thrombosis in patients with COVID-19, thereby maintaining persistent viral infections. However, characteristics and function mechanisms of these lncRNAs in COVID-19 still remain obscure. This review will focus on the roles of lncRNAs in COVID-19 infection and antiviral responses and underlying regulatory mechanisms as well as its application prospects and challenges.

## Pathogenesis of SARS-CoV-2

Coronaviruses (CoVs) are enveloped single-stranded positive-sense RNA viruses, which feature the largest viral RNA genomes (approximately 28-32 kb) with a 5’ cap and a 3’ polyadenylated tail and belong to the Coronaviridae family of the order Nidovirales (11, 12). The CoV genome can be roughly divided into 6 or 7 regions, each of which contains at least one open reading frame (ORF). The first reading frame, which comprises approximately two-thirds of the genome, encodes replicases, while the rest mainly encode structural proteins, generally including the spike (S), nucleocapsid (N), membrane (M), and small envelope (E) proteins. In addition, a few CoVs have hemagglutinin esterase (HE) glycoproteins, which play various roles in viral entry and transmission (11, 13, 14). The S glycoprotein contains the main CoV antigen and is mainly responsible for host cell adhesion, erythrocyte agglutination and membrane fusion during the early stage of CoV infection; the N nucleoprotein is located in the core of the virus particle and is mainly responsible for the replication of viral genomic RNA. It binds to the viral genome to recognize the signals that package the enveloped genome into virus particles. The E protein is mainly involved in the assembly and release of virions. The M protein is the most abundant structural protein in CoVs and contains three transmembrane domains, which interact with the E protein to mediate the assembly of the viral envelope. Based on their genomic structural characteristics, CoVs are divided into four genera: Alphacoronavirus, Betacoronavirus, Gammacoronavirus, and Deltacoronavirus (15–17). To date, seven human coronaviruses (HCoVs) have been identified, namely, HCoV-NL63, HCoV-229E, HCoV-OC43, HCoV-HKU1, severe acute respiratory syndrome coronavirus (SARS-CoV), Middle East respiratory syndrome coronavirus (MERS-CoV) and SARS-CoV-2 (16, 17).

As the main pathogen of COVID-19, SARS-CoV-2 has been identified as a betacoronavirus through whole-genome sequencing and phylogenetic analyses of lower respiratory tract samples from patients (18); SARS-CoV and MERS-CoV are also betacoronaviruses, and the SARS-CoV-2 genome is 45-90% similar to that of SARS-CoV. However, the key spike genes (encoding the S protein) that interact with host cells are significantly different among CoVs (19–21). Current evidence suggests that bats are the original host of SARS-CoV-2, but the intermediate host has not yet been determined (19). The S proteins of SARS-CoV and MERS-CoV infect human alveolar epithelial cells by interacting with the human angiotensin-converting enzyme 2 (ACE2) protein and DPP4 protein, respectively (22). The S protein contains two subunits (S1 and S2), and the N- and C-termini of S1 can be divided into two independent domains—the N-terminal domain (NTD) and the C-terminal domain (CTD)—both of which can serve as receptor-binding domains (22). The latest research shows that the amino acid sequences of the SARS-CoV-2 and SARS-CoV RNA-binding domains (RBDs) are highly similar to the predicted protein structures, suggesting that SARS-CoV-2 invades host cells *via* ACE2 receptor (19, 20). A recent study found that the S protein of SARS-CoV-2 strongly interacts with human ACE2 expressed by alveolar epithelial cells and can increase vascular permeability (23). In addition, the binding affinity of SARS-CoV-2 envelope spikes for the cellular ACE2 receptor is 10-20-fold higher than that of the corresponding structures of SARS-CoV, which may account for the high infectivity of the SARS-CoV-2 in humans (24).

**Figure 1:**
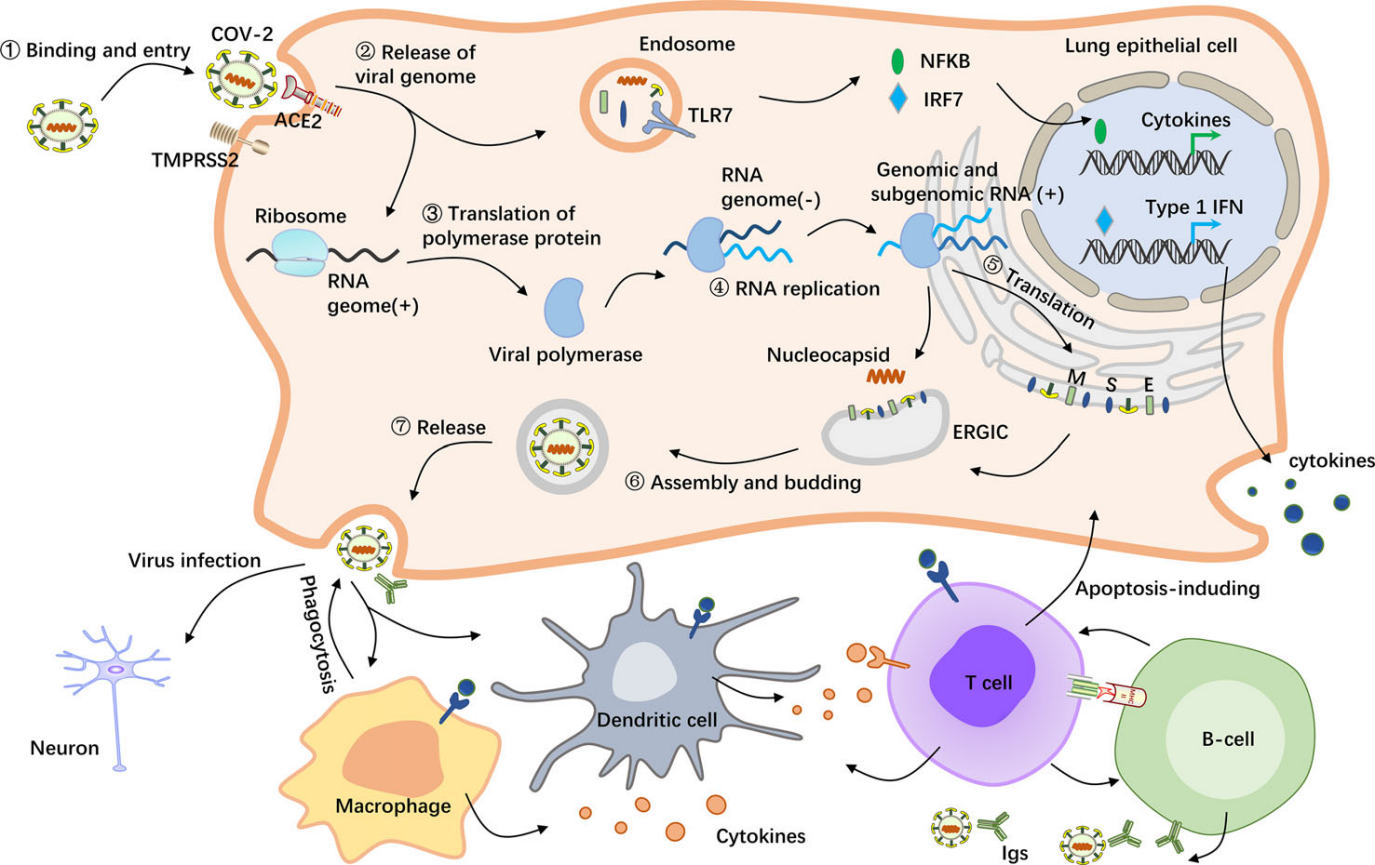
Schematic representation of the pathological mechanism of COVID-19. SARS-CoV-2 enters host cells by first binding to angiotensin-converting enzyme 2 (ACE2) *via the* surface spike (S) protein. Furthermore, effective host cell entry depends on cleavage of the S1/S2 site by the surface transmembrane protease serine 2 (TMPRSS2) (26, 27). Following entry of the virus into the host cell, viral genomic RNA is released into the cytosol, where it is translated into viral polymerase proteins. Here, subgenomic (–) RNAs are synthesized and used as templates for subgenomic (+) messenger RNAs (mRNAs). The nucleocapsid (N) structural protein and viral RNA are replicated, transcribed, and synthesized in the cytoplasm, whereas other viral structural proteins, including the S protein, membrane (M) protein and envelope (E) protein, are transcribed and then translated in the endoplasmic reticulum (ER). The resulting structural proteins are inserted into the ER-Golgi intermediate compartment (ERGIC) for virion assembly, followed by release of the nascent virion from the host cell *via* exocytosis. Subsequently, the resulting massive number of virus particles first activate innate immune cells such as macrophages and dendritic cells, which can not only kill the infected cells but also control the viral replication in the cells by secreting cytokines and activating more immune cells to reach the infected site (28). In this process, B cells produce and secrete numerous antibodies that bind to the virus, thereby blocking the virus from entering the body and enhancing viral phagocytosis by phagocytes. In addition, the nucleic acid substance RNA released from the virus will be recognized by the pattern recognition receptor TLR7, which is located on the endosomal membrane surface and can recognize RNA (29). After activation of TLR7 by viral RNA, multiple proteins are recruited to form a complex, which promotes the transfer of transcription factors such as NF-kB and IRF7 to the nucleus and activates the expression of proinflammatory cytokines, which regulate viral clearance by the host immune system (30, 31).

Amid the progress of infection, although most symptoms of COVID-19 are mild in most patients, some are initially mild and then suddenly exacerbate at a later time point, leading to death from multiple organ failure (MOF), which may be caused by cytokine storms (25). Upon human infection with SARS-CoV-2, the S protein binds to ACE2 receptor on the cell surface, followed by priming by a host proteinase, the most important of which is transmembrane serine protease 2 (TMPRSS2), to stimulate viral entry into the host cell (26, 27). After entering alveolar epithelial cells, SARS-CoV-2 consumes stored energy and substances, including nucleotides, amino acids and lipids, by hijacking the cell’s replication and translation machinery, resulting in massive viral replication in a short time and ultimately leading to cellular collapse. However, bodily cells protect themselves from destruction by building strong immune defenses against the virus. On the one hand, macrophages in the immune system phagocytose entire viral particles and hydrolyze the virus with various hydrolytic enzymes in the lysosomes (28). On the other hand, if the virus escapes elimination and releases its RNA, it will be recognized by the pattern recognition receptor toll-like receptor (TLR) 7, which is located on the surface of the endosomal membrane and can recognize RNA (29). After activation of TLR7 by viral RNA, multiple proteins are recruited to form a complex, which promotes the transfer of transcription factors (TFs), such as NF-kB and IRF7, to the nucleus and activates the expression of proinflammatory cytokines (30, 31). The pathological role and the regulatory mechanism of COVID-19 on lung epithelial cell is shown in **Figure 1**. These cytokines can induce the host’s immune system to clear the virus; however, if the immune system is overactivated, the cytokines will infiltrate the lung tissue and develop a cytokine storm pathology, which may lead to the progression of acute respiratory distress syndrome (ARDS), MOF and even death (32). In addition to alveolar epithelial cells, both human monocytes and human macrophages express ACE2 and can be directly infected with SARS-CoV-2, thereby increasing the transcription of proinflammatory genes associated with COVID-19 severity. Moreover, a recent review of the literature on this topic revealed that the neuropilin-1 (NRP1) receptor on host cells can also bind to the S protein of SARS-CoV-2, thereby enhancing the ability of the virus to infect host cells (33).

## Regulation of LncRNAs During The SARS-CoV-2 Life Cycle

LncRNAs are endogenously expressed non-coding RNAs (ncRNAs) with a length of more than 200 nucleotides. An increasing number of studies have demonstrated lncRNAs as a new class of regulatory molecules that mediate host-virus interactions. LncRNAs induced by viruses have been reported to regulate innate immune responses to eliminate viral infections *via* different mechanisms (34). In order to understand the effect of lncRNAs on SARS-CoV-2, we discussed recent research progress on ncRNAs involved in the life process of the virus.

### Viral Gene Expression

After SARS-CoV-2 entering the host cell, the viral genome is released into the cytoplasm, followed by elaborate integration and replication of the viral genome and that releases new progeny virions to infect other host cells. The number of lncRNA transcripts produced by human cells is estimated to be approximately 200,000, and the rich sequence information and structural potential inherent in host lncRNA populations, whether directly regulated or indirectly regulated through their protein ligands, are sufficient to support the regulation of cellular processes and the viral life cycle to establish persistent infection (35, 36). Josset L et al. constructed a mouse model of SARS-CoV infection for high-throughput sequencing analysis of the lungs, and a total of 5329 differentially expressed lncRNAs were found (37). In this study, the lncRNA MALAT1 was downregulated and highly negatively correlated with genes that encode 60S ribosomal protein (RPL6), endoplasmic reticulum protein retention receptor 3 (KDELR3) and tubulinα1 (TubA1A), while the lncRNA NEAT1 was upregulated and enriched in pathways related to the viral defense response, innate immune response, and inflammatory response. Due to the apparent similarity between SARS-CoV-2 and SARS-CoV, it can be speculated that lncRNAs have differential transcription in response to SARS-CoV-2 infection. Meanwhile, a large number of abnormal expressions of lncRNAs were found in bronchoalveolar lavage fluid (BALF) and peripheral blood mononuclear cells (PBMC) from COVID-19 patients in recent studies, which further verified this idea (8).

Moreover, multiple studies based on bioanalysis have identified various dysregulated lncRNAs associated with SARS-CoV-2 replication. For example, differential expression of MALAT1, found in COVID-19 patients, has previously considered to be a regulator of gene expression that encodes markers of lung cancer metastasis, and its deletion leads to the activation of P53 and its target genes, which affect the normal cell cycle (38). However, recent studies found that MALAT1 can reduce the epigenetic silencing of viral transcription by regulating promoter-enhancer interactions to promote viral transcription and infection (39). In addition, other studies have shown that lncRNA NEAT1 controls RNA regulatory processes by forming nonmembranous organelle nuclear paraspeckles (40). Knockout of NEAT1 can enhance viral production by promoting the export of HIV-1 mRNA from the nucleus to the cytoplasm in HeLa cells (41).

### Antiviral Innate Immune Signaling and Cytokine Productions

Antiviral immune response is associated with both innate and adaptive immune responses. The regulatory effects of lncRNAs involving signal pathways in anti-CoV immune response has been shown in **Table 1**. Influenza viral RNA is recognized by the host’s pathogen recognition receptors after infection, which can activate the antiviral natural innate immune response. As the first line of defense against viral infection, type I interferons (IFN-I, including interferon (IFN)-α and IFN-β) play a central role by activating the expression of hundreds of IFN-stimulating genes (ISGs) (50). Interestingly, the innate immune response of SARS-CoV-2 also can be regulated by lncRNA through association with IFN mechanism pathway. A recent study using longitudinal total transcriptome RNA sequencing in patients with COVID-19 found that IFN-1 response was significantly downregulated in the ncRNA regulatory network (43). Moreover, previous studies revealed an lncRNA NRAV that negatively regulates the transcriptional initiation of several key ISGs, including IFITM3 and MXA, through histone modification. In human lung epithelial cells infected by influenza A virus (IAV), the expression of the lncRNA NRAV was decreased significantly; overexpression of NRAV promoted viral replication and increased virulence, while silencing NRAV significantly inhibited viral replication and virulence (51). NRAV negatively regulates the transcriptional initiation of several key ISGs, including IFITM3 and MXA, through histone modification, suggesting that decreased NRAV expression is part of the host innate immune response. In addition, as mentioned above, lncRNA NEAT1 was enriched in virus defense response and innate immune response in SARS-CoV-infected mice (37). Since several studies have shown that influenza virus and SARS-CoV have similar expression patterns in the lung tissues of infected mice, coupled with the high similarity between SARS-CoV and SARS-CoV-2 (52), it is boldly speculated that lncRNAs may have the potential to prohibit SARS-CoV-2 infection by regulating IFN.

**Table 1:** Regulatory effects of lncRNAs pathways in anti-CoV immune response.

Human coronavirus	Signal pathways	Target	lncRNAs	Function	Immune reaction	References
SARS-CoV-2	IFN	IFN- γ	LINC02384	Antiviral response	Innate Immune	(8)
		IFN-I	EGOT	Antiviral response	Innate Immune	(42)
	NLRP3	CAPN1	MALAT1, NEAT1	Inflammation	Innate and adaptive immune	(8)
	IL-17	IL-17	AL392172, HOTAIRM1, PVT1	Viral transcription and Inflammation	Innate and adaptive immune	(8)
	Cytokine	IL-6, TNF-α	WAKMAR2, EGOT, EPB41L4A-AS1, ENSG00000271646	Immune and inflammatory responses	Innate and adaptive immune	(42)
	Th17 cell differentiation	SATB1, LEF1, IL6ST, CCR7	CCR7-AS-1, LEF1-AS-1, LINC-CCR7-2, LINC-TCF7-1, TCF7-AS -1	T cell activation and differentiation	Adaptive immune	(43)
	MAPK	AP-1	LINC-JUND-1, LINC-HSP90AA1-1, HIF1A-AS-1, RORA-AS-7, RORA-AS-8	T cell activation and differentiation	Adaptive immune	(43)
	AKT, PAK, ERK	IL-6	NORAD, RAD51-AS1	Cytokine storm and Inflammation	Innate and adaptive immune	(44)
		TNFα	NORAD, RAD51-AS1, GAS5	Cytokine storm and Inflammation	Innate and adaptive immune	(44)
	Akt/mTOR	TNFRSF1B, FCGR2A	MSTRG.119845.30	Sustained T cell and B cell responses	Adaptive immune	(45)
		STAT3	MSTRG.106112.2	Block virus autophagy and apoptosis	Innate and adaptive immune	(45)
	NF-κB	IL-6, TNF, NF-κB1	MALAT1, WAKMAR2, EGOT, DANCR, NONHSAT122723.2	Inflammation	Innate and adaptive immune	(8, 42, 46, 47)
	P13K/AKT	IL1RN	ENST00000631362	Inflammation	Innate and adaptive immune	(47)
SARS-CoV	IFN	ISGs	NEAT1, Adapt33	limit viral replication within the cells	Innate Immune	(37)
		IFN-α	Gm26917	Antiviral response	Innate Immune	(48)
	TGF-β	SMAD2, SMAD3, SMAD4, SMAD7, and TGFBR1	SNHG1, SMC2-AS1	Promote virus infection	Innate and adaptive immune	(49)

Remarkably, after recognizing viral components, TLR can not only produce IFN, but also promote the release of inflammatory cytokines. In addition, the presence of neutrophils and macrophage cluster-1 has been shown to be a hallmark of severe COVID-19, and their activation also leads to excessive proinflammatory cytokine responses. Of note, several lncRNA can directly regulate the transcription of cytokines. Recently, Oranrewaju B. et al. identified 22 lncRNAs that target 10 cytokines overexpressed in COVID-19 cytokine storms (44). In particular, the lncRNA NORAD, activated by DNA damage, targets five of the ten cytokines in cytokine storms (interleukin (IL)-6, IL-10, CSF3, tumor necrosis factor (TNF)-α and CXCL10), which is more than other identified lncRNAs. All of these significant cytokines are involved in three pathways: PEDF-induced signaling, cytokine signaling in the immune system, and signaling in the innate immune system. The results of this study sufficiently demonstrated that lncRNAs play critical roles in COVID-19.

The adaptive immune response caused by virus mainly consists of B cell-mediated humoral immunity response and T cell-mediated cellular immunity response. As early as September 2020, the severity of COVID-19 disease was reported to be associated with SARS-CoV-2 virus-specific antibody response (53). Moderate severe patients can induce a strong humoral immune response and produce a higher antibody level. Actually, a part of antibodies called neutralizing antibodies can recognize the RBD region of novel coronavirus protein and bind to it, thus preventing SARS-CoV-2 from binding to ACE2 on human cells and blocking viral infection. On the other hand, asymptomatic and mild patients have low antibody levels but good virus-specific immunity to CD4+ T and CD8+ T cells that provide long-term protection. In clinical analysis of patients with COVID-19, inflammatory cytokines and chemokines have been shown to upregulate the activated CD4+ T cellular response (54). Due to the interaction between lncRNA and inflammatory cytokines, the T cell immune response of the virus is likely to be dependent on the regulation of lncRNA. Recent research supports this idea. For example, Zheng HY et al. showed that a mass of upregulated differentially expressed lncRNAs positively regulated the differentiation of lymphocytes and T cells at the convalescence stages, which are closely linked with inflammatory factor genes TNF and IL1B (43). Likewise, another high-throughput sequencing of COVID-19 also revealed significant enrichment of lncRNAs in T cell-related pathways (45). Currently, there are no studies have shown the regulatory effect of lncRNA on viral antibody production. However, the significant correlation between the neutralizing antibody titers and the numbers of nucleocapsid protein (NP)-specific T cells, suggesting the correlation between lncRNA and humoral immune response (55).

### Virus Immune Evasion

During the evolution of the organism to resist pathogenic microorganisms for survival, the immune function is developed, while virus developed the mechanism to evade the immune surveillance of the host through long evolutionary selection. Correspondingly, SARS-CoV-2 employs immunopassivation or delay to evade or weaken host immune clearance, allowing them either continue replication or overactivation of the inflammatory response. LncRNAs are extremely important effector molecules in the body’s antiviral immunity, and the understanding of immune evasion of SARS-CoV-2 will provide new insights on the role of lncRNAs in viral immune regulation.

In the early stage of SARS-CoV-2 infection, the innate antiviral immune response of the body was inhibited, leading to the delayed production of IFN, especially IFN-β. This results in the appearance of asymptomatic patients. For example, Lei X et al. showed that the expression of IFN-β was barely induced early during viral infection, while surged at late time point, suggesting that the antiviral response of SARS-CoV-2 to the host was weakened (56). Further analysis showed that ORF6, an accessory protein gene of the virus, could inhibit IFN-β production at the level of or downstream of interferon regulatory factor (IRF)-3 activation, and that the C-terminal region of ORF6 was the core of its antagonism. Another study showed that SARS-CoV-2 N protein is involved in IRF3 phosphorylation and nuclear translocation (57). Moreover, they found that SARS-CoV-2 N protein represses IFN-β production by interfering with the retinoic acid-induced gene I (RIG-I) pathway. Furthermore, antigenic variation is another effective mechanism as an innate immune evasion. As mentioned earlier, the lncRNA network of COVID-19 patients showed significantly downregulated IFN-I response, while the immune evasion of the virus was related to IFN (43). Therefore, SARS-CoV-2 may achieve innate evasion escape through the above mechanism of lncRNAs mediated IFN regulation.

SARS-CoV-2 can also trigger the release of cytokines in cellular avoidance immunity, which promotes T-cell depletion (50). In addition, it has been found that for SARS-CoV, which is similar to SARS-CoV-2, infection of immature dendritic cells showed low expression of costimulatory cell surface molecules (CD80/86) and human leukocyte antigen class II (HLA-II) molecules and interferes with viral antigen presentation, thus affecting humoral immunity (58). Although lncRNAs that control adaptive immune escape of viruses have not been clearly defined, some lncRNAs implicated in immune cells may be contributory.

## Potential Mechanisms of LncRNAs in COVID-19

Most patients with SARS-CoV-2 have asymptomatic, mild or moderate disease, but approximately 15-20% develop severe pneumonia, and ARDS is evident in approximately 5% (59, 60). Furthermore, several critical patients develop secondary coagulation dysfunction to form deep venous thrombosis of the lower extremity. Notably, the leading causes of death in COVID-19 patients are respiratory failure, septic shock, heart failure, bleeding, and renal failure (61, 62). Triggering of the host immune may lead to differential expression of both viral and host lncRNAs, as mentioned previously, which may have important impacts on the tissues and organs of the patient (63).

### Inflammatory Response

Patients with COVID-19 presented inflammation-related parasympathetic complications and post-infection manifestations that can lead to respiratory failure and multiple organ failure (4, 62, 64). Serological tests show that the inflammatory cytokine storm response (ICSR) is associated with COVID-19 severity and mortality as a result of an uncontrolled systemic inflammatory response in the host (4, 64–66). IL-6 and NLRP3 inflammasome complexes are the main immune components of the immune response after pathogen infection, and their function and expression are significantly affected by ncRNAs, including lncRNAs (44, 67, 68). IL-6, which is a typical proinflammatory factor causing cytokine storm, seems have a direct association with a worsened patient condition, is particularly affected (69, 70). Actually, both the IL-6 and NLRP3 signaling pathways are associated with activation of the nuclear factor κB (NF-κB) pathway (71–73). Beyond that, it has been reported that lncRNAs are directly involved in the regulation of the NF-κB signaling pathway, resulting in cytokine storms during severe COVID-19 infection (42, 45). Of course, some lncRNAs can also be regulated by IL-6. Shang W et al. identified a lncRNA pseudogene, olfr29-ps1, that is expressed in myeloid-derived suppressor cells (MDSCs) and upregulated by the proinflammatory cytokine IL6 (74).

In addition, Pengfei Zhang et al. found that NEAT1 is associated with the NLRP3, NLRC4 and AIM2 inflammasomes in mouse macrophages, not only promoting inflammasome assembly and subsequent pro-caspase-1 processing but also stabilizing mature caspase-1 to promote IL-1 generation and focal disintegration (75). In mouse models of peritonitis and pneumonia, NEAT1 deficiency significantly reduced the inflammatory response. These results reveal the role of lncRNAs in innate immunity and suggest that NEAT1 is a common mediator of inflammatory signaling induction. Furthermore, Chanan Meydan et al. showed that sharp declines in the lung pathology-associated lncRNA DANCR and the nuclear paraspeckles forming the neuroprotective lncRNA NEAT1 are potentially associated with the susceptibility to and outcome of COVID-19 infection (46). The inflammation-modulating lncRNA DANCR, as a potential chief regulator, is responsible for the surveillance of inflammatory cholinergic blockade, thus supporting the maintenance of a delicate balance between pro-inflammatory and anti-inflammatory pathways in SARS-CoV-2-affected lung and brain tissues. Of note, NEAT1 interacts with inflammatory pathways, leading to the transcriptional activation of IL-8 (76). NEAT1 is further implicated in inflammation by its association with regulators of inflammasome-associated proteins and by its assembly with other molecular elements into an immune-controlling ribonuclear complex (75, 77). Meanwhile, their findings indicate that DANCR and NEAT1 contribute to the inflammation-regulating ncRNA-mRNA network and operate in conjunction with other coding and noncoding mediators (78). Other studies utilized bioinformatic approaches to elucidate the interactions among SARS-CoV/human proteins, miRNAs, and lncRNAs and speculated that SARS-CoV infection in mammals increases the inflammatory and immune responses by directly interacting with host proteins (49). This research provides arguments to consider that ncRNA-mRNA network. The interconnectivity of this network may be elucidated upon examining shared components such as miR-135b-5p, miR-656-3p, and miR-19a-3p, as some may interact with key relevant downstream mediators such as TNF and IL-6 to affect inflammation, leading to COVID-19 (46).

It is noteworthy that inflammatory cytokine imbalance can not only cause severe tissue damage, but also interact with the coagulation system, resulting in hypercoagulability and promoting thrombosis (79). Similarly, the clotting disorder in COVID-19 patients may be related to inflammatory cytokines (80). At present, the specific mechanism of COVID-19-related coagulation disorders has not been fully elucidated, so understanding the role of lncRNA in virus-induced inflammatory response will be helpful to the study of its pathogenesis.

### Neurological Sequelae

To date, various neurological sequelae of COVID-19 have been reported. These diseases include encephalitis, demyelinating entities (myelitis and encephalomyelitis), encephalopathy without cerebral inflammation, epilepsy, polyradiculopathy, and cerebrovascular manifestations (81, 82). More commonly, in a European study of 417 COVID-19 patients, approximately 86% of patients with mild to moderate disease reported alterations of smell and taste (83). Following reports of involuntary breathing, shortness of breath and loss of attention in COVID-19 patients, scientists began to speculate that SARS-CoV-2 not only affects the lungs but also seriously affects neurons, especially those in the medulla oblongata that regulate respiration and lung and heart function. Researchers have also confirmed the presence of SARS-CoV-2 in cerebrospinal fluid *via* gene sequencing, thereby backing up the abovementioned hypothesis (84). Notably, approximately 40% of the lncRNAs detected to date are specifically expressed in the brain (85). Mercer identified 849 lncRNAs in the brains of adult mice using high-throughput microarray technology, and most of them were specifically distributed in brain anatomical regions, cell types and suborganelles (86).

**Figure 2:**
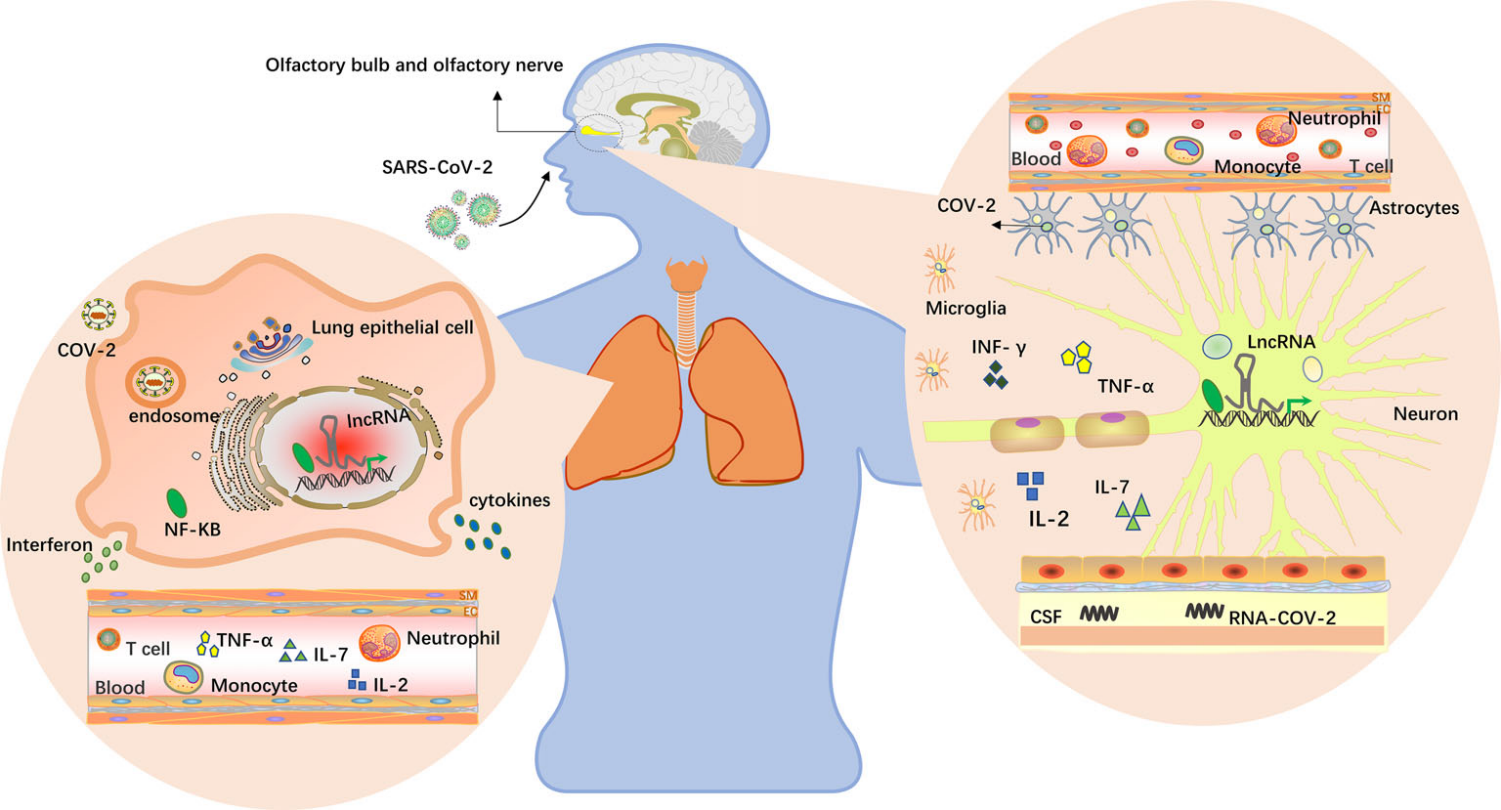
LncRNAs in the brain and lungs. The virus enters the body through the nose and mouth, causing pneumonia and other symptoms. Upon oral infection, the virus reaches the lungs and, from there, can spread into the CNS by the hematogenous route. Upon nasal infection, SARS-CoV-2 can reach the CNS through the olfactory bulb (89). Viruses have been detected in the CSFs of infected patients, and inflammation in the brain has been described. Multiple studies have found that lncRNAs are differentially expressed in humans, and lncRNAs near the alveoli and olfactory bulb may be induced by viruses to participate in specific cellular activities in this region, including virus infection, replication, latent infection and immune responses. Cytokine storms occur when the immune system is overactivated, causing a surge in interleukin-2, IL-7, interferon-γ and tumor necrosis factor expression (64, 90).

There were many lncRNAs distributed in the olfactory bulb and cerebellum of the hippocampal cortex, showing significant specificity. By regulating the expression of some important coding genes, lncRNAs enable the nervous system to grow and develop in a certain space and at a certain time and participate in the implementation of nervous system function (87). Guan et al. identified a lncRNA by pseudorabies virus (PRV) transcriptome sequencing and studied its expression in nerve cells; they found that the DNA sequence of latent PRV in the host’s central nervous system between ep0 and ie180 encoded several latency-associated transcripts (LATs), all of which were lncRNAs, and some of the associated lncRNAs were expressed at low levels in PRV-infected epithelial cells (88). Some of the viral-encoded lncRNAs were knocked down by siRNA, which significantly promoted viral replication. Additionally, Das G et al. reported that SARS-CoV-2 may enter the central nervous system through the olfactory bulb rather than the common hematological route of entry into the central nervous system through the nose (89), as shown in **Figure 2**. The olfactory nerve connects to nasal epithelial cells and the olfactory bulb channels of the central nervous system. Therefore, the virus might enter the olfactory mucosa, mostly consisting of olfactory neurons, blood vessels and epithelial cells. The olfactory mucosa is connected to the olfactory bulb through the cribriform plate, which is found at the very base of the frontal lobe of the brain (91). Moreover, PRV showed neurophagocytosis and latency in hosts in which it naturally infected, similar to SARS-CoV-2. Furthermore, using GO and KEGG enrichment analysis of COVID-19 patients, recent studies have found that differential lncRNAs play significant roles in neurons, lung cancer, and many other aspects (47). Therefore, latent SARS-CoV-2 in the host’s central nervous system is thought to not only encode relevant lncRNAs but also to participate in special nerve cell activities in the region by regulating the expression of lncRNAs near the host’s olfactory bulb, and these phenomena are potentially related to infection, replication, nerve cell invasion and latent viral infection.

**Figure 3:**
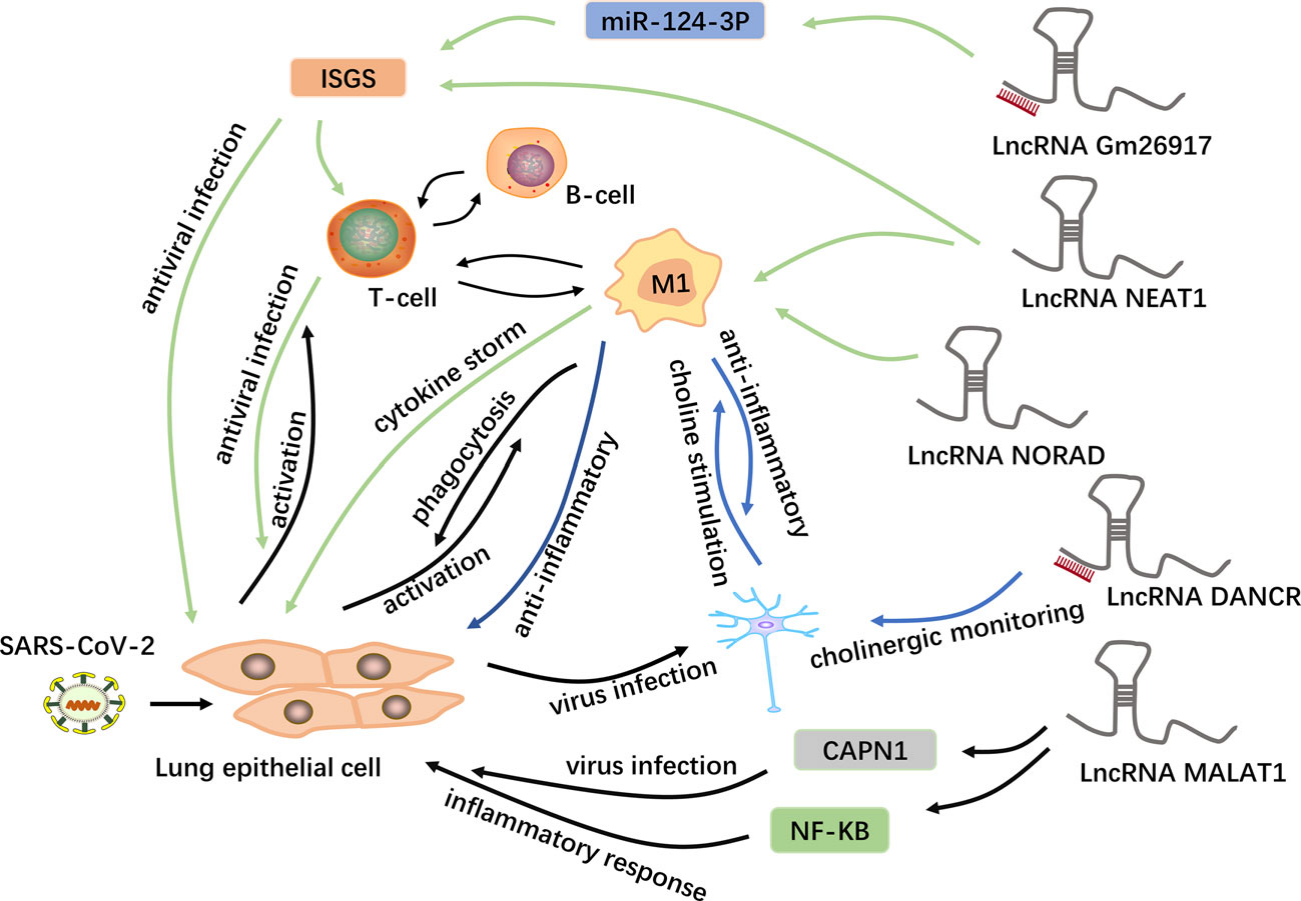
Potential mechanisms of lncRNAs in COVID-19. LncRNAs are a new class of regulatory factors that mediate host-virus interactions. The lncRNA GM26917 acts as an “miRNA sponge” and interacts with miR-124-3p to regulate the expression of IFN-stimulating genes (ISGs) (48). On the one hand, the epithelial cells themselves produce type 1 interferons to resist viral infection; on the other hand, the activated immune cells release interferons in large quantities to control the viral replication in the cell. In addition to regulating the expression of ISGs, the lncRNA NEAT1 is also associated with the NLRP3, NLRC4 and AIM2 inflammasomes in macrophages, thus participating in the induction of inflammation (93). The lncRNA NORAD can target five of the ten cytokines involved in cytokine storms and is thus involved in cytokine storms (44). In addition, the lncRNA MALAT1 is highly likely to regulate lung inflammatory injury caused by cytokines through the NF-kB, and can also target and down-regulate CAPN1, participating in viral infection (8, 93–95). Moreover, some viruses can spread from the lungs to the central nervous system *via a* blood-borne route. The lncRNA DANCR is potentially the chief regulator responsible for the surveillance of inflammatory cholinergic blockades and thus plays distinct roles in pneumonia and neuroinflammation (46). Acetylcholine interacts with macrophages to inhibit the synthesis of inflammatory cytokines.

Additionally, neuroinflammation, also caused by cytokine storms, is mainly manifested by the secretion of IL-6 by macrophages, and secreted IL-6 interacts with lncRNAs, as mentioned earlier (90, 92). In combination with other previous studies, these findings demonstrate the potential mechanism by which lncRNAs having important regulatory roles in COVID-19, as shown in **Figure 3**. However, the specific regulatory details and mechanisms are still unclear and require further investigation.

## The Application Prospects of LncRNAs in Diagnosis and Treatment

The COVID-19 pandemic is an ongoing challenge in the medical and research community. At present, SARS-CoV-2 has been transmitted rapidly in many countries and continues to spread among people, causing serious disease. There is thus an urgent need for the development of effective preventive and therapeutic strategies for SARS-CoV-2 outbreaks. In recent years, many scholars have performed research on lncRNAs and diseases, especially virus, and have made significant progress. LncRNAs have not only become promising candidates for the treatment of a variety of diseases but have also been identified to play a powerful potential role in host-virus interactions (96), which provides new ideas for research on treating and/or preventing COVID-19. This paper summarizes the involvement of lncRNAs in the regulation of SARS-CoV-2 (as shown in **Table 2**).

**Table 2:** Significant lncRNAs associated with COVID-19.

Human coronavirus	lncRNAs	Regulate direction	Origin	Organism/organ/cell type	Targets regulation	Biological effect	Reference
SARS-CoV-2	AC009088	Up-regulation	Bioinformatics prediction	Peripheral blood mononuclear cells (PBMC)	Downregulation Pycard	Inhibit Pycard transcription and fine-tune the inflammatory process	(8)
	LINC02384	Down-regulation	Bioinformatics prediction	PBMC	Regulated IFN- γ	Regulated innate immune response	(8)
	AL392172	Up-regulation	Bioinformatics prediction	Bronchoalveolar lavage fluid (BALF)	Regulated IL-17 signalling pathway and Nonsense- Mediated Decay (NMD) pathway	Regulated viral transcription and inflammatory development	(8)
	HOTAIRM1	Down-regulation	Bioinformatics prediction	BALF	Regulated IL-17 signalling pathway and Nonsense- Mediated Decay (NMD) pathway, restricting the ORF1ab	Regulated viral transcription and inflammatory development	(8)
	PVT1	Up-regulation	Bioinformatics prediction	BALF	Regulated IL-17 signalling pathway and Nonsense- Mediated Decay (NMD) pathway, restricting the ORF1ab	Regulated viral transcription and inflammatory development	(8)
	SNHG25	Down-regulation	Bioinformatics prediction	BAL cells	Induced TNF-α, IL-6, and IL-1, as well as Ccl2, Ccl3, and Cxcl10 inflammatory chemokines	Regulated neutrophils chemotaxis and reduce inflammation injury	(9)
	MALAT1	Down-regulation	Bioinformatics prediction	BAL cells	Induced TNF-α, IL-6, and IL-1, as well as Ccl2, Ccl3, and Cxcl10 inflammatory chemokines	Regulated neutrophils chemotaxis and reduce inflammation injury	(9)
	NEAT1	Down-regulation	Bioinformatics prediction	BAL cells	Induced TNF-α, IL-6, and IL-1, as well as Ccl2, Ccl3, and Cxcl10 inflammatory chemokines	Regulated neutrophils chemotaxis and reduce inflammation injury	(9)
	WAKMAR2	Up-regulation	Bioinformatics prediction	BALF	Regulated cytokine signaling pathway	Promote virus replication	(42)
	EGOT	Up-regulation	Bioinformatics prediction	BALF	Regulated cytokine signaling pathway	Promote virus replication	(42)
	HIF1A-AS-1	Up-regulation	Bioinformatics prediction	PBMC	Regulated the interaction between gene HIF1α and Jun	Enhanced the effector function of T cells and promote virus clearance	(43)
	RORA-AS-7	Up-regulation	Bioinformatics prediction	PBMC	Regulated the interaction between gene RORA and AP-1	Controlled T cell differentiation	(43)
	GAS5	Up-regulation	Bioinformatics prediction	Unknown	Increased IL-10, TNFα	Inhibited its promoting activity against the virus and reduce lipopolysaccharide inflflammatory injury	(44)
	NRAV	Down-regulation	Bioinformatics prediction	Unknown	Increased CCL3,CCL2	Promoted the host immune response and suppresse virus replication and virulence	(44)
	TUG1	Up-regulation	Bioinformatics prediction	Unknown	Increased IL-7,CCL2	Promoted inflammation and infection	(44)
	NORAD	Up-regulation	Bioinformatics prediction	Unknown	Increased CXCL10, CSF3, IL-6, TNFa	Led to cytokine storm	(44)
	RAD51-AS1	Up-regulation	Bioinformatics prediction	Unknown	Increased CCL2, IL-6, TNFα	Promoted pro-inflammatory immune response,resulting in cytokine storm	(44)
	MSTRG.119845.30	Unknown	Bioinformatics prediction	PBMC	Regulated TNFRSF1B, FCGR2A	Regulated adaptive immunity and inflammation	(45)
	MSTRG.106112.2	Unknown	Bioinformatics prediction	PBMC	Regulated STAT3	Blocked virus autophagy and apoptosis	(45)
	DANCR	Down-regulation	Experiment validation	Inflammation-prone lung tissues	Increased REL, RELA, and NFkB1 and to AChE and IL-1b	Promoted infection	(46)
	NONHSAT122723.2	Up-regulation	Bioinformatics prediction	PBMC	Down-regulated NF-κB	Regulated immune and inflammatory responses	(47)
	ENST00000631362	Down-regulation	Bioinformatics prediction	PBMC	Down-regulated IL1RN	Involved in inflammation	(47)
	NEAT1	Up-regulation	Experiment validation	Normal human bronchial epithelial(NHBE) cells, BALF	Down-regulated CAPN1	Involved in inflammation	(8, 93–95)
	MALAT1	Up-regulation	Experiment validation	NHBEC, BALF	Down-regulated CAPN1	Involved in inflammation	(8, 93–95)
	TTTY15	Up-regulation	Bioinformatics prediction	COVID-19 infected lung tissue	Promoted T-box transcription factor 4 (TBX4)	Inhibited cell infection	(97, 98)
	TPTEP1	Up-regulation	Bioinformatics prediction	COVID-19 infected lung tissue	Suppressed STAT3 phosphorylation	Promoted cell survival and inhibite infection	(97, 99)
	GATA5	Up-regulation	Bioinformatics prediction	PBMC	Suppressed ACE2 gene	Prevented virus from infecting cells	(100)
SARS-CoV	MALAT1	Down-regulation	Experiment validation	Mice lung	Up-regulated RPL6, KDELR3,Tuba1a	Promoted host cell proliferation and production of viral particles	(37)
	NEAT1	Up-regulation	Experiment validation	Cast/EIJ and Nzo/HILT mice lung	Up-regulate ISGs,	Sequester some virus mRNAs and promote defense response to virus	(37)
	Adapt33	Up-regulation	Experiment validation	WSB/EIJ mice lung	Promoted Hspa9 and Myc	Enhanced host cell apoptosis and Inhibition of viral replication	(37)
	SNHG1	Up-regulation	Bioinformatics prediction	Unknown	Reduce CCL2 and induced TGF-β	Reduced immune response and promote virus infection	(44, 49)
	SMC2-AS1	Up-regulation	Bioinformatics prediction	Unknown	TGF-β,Wnt	Lung Repair and Regeneration	(49, 101–103
	ROR1-AS1, FTX	Up-regulation	Bioinformatics prediction	Unknown	Promoted MAPK, ERBB	Promoted the entry of the virus into the host cell	(49, 104)
	Gm26917	Up-regulation	Bioinformatics prediction	Unknown	Sponged miR-124-3p	Promoted interferon signalling and antiviral-mechanism	(48)

### LncRNA as a Diagnostic Biomarker

Previous studies have confirmed that lncRNAs are differentially expressed in patients with SARS-CoV-2, which are closely related to the occurrence and development of the disease. Cheng J et al. reported altered lncRNAs between severe COVID-19 patients, non-severe COVID-19 patients and healthy controls (100). The findings showed that lncRNA GATA5 was significantly elevated in severe condition, indicating that the expression level of GATA5 was positively correlated with COVID-19 severity. Moreover, another study also found that lncRNA DANCR and NEAT1 are associated with genes that distinguish between mild and severe damage of SARS-CoV-2 (46). It implies the potential role of these two lncRNAs in risk stratification, suggesting that the abnormal expression level of lncRNAs may provide a new reference index for the diagnosis and prognosis of COVID-19.

In addition, the differential expression of lncRNA may reflect the physiological and pathological changes of human body. For instance, lncRNAs NEAT1 and MALAT1, were significantly upregulated in BALF samples from patients compared to healthy samples, which have been shown to be potential biomarkers for HIV infection (8, 105). The further investigation revealed that both lncRNAs were negatively correlated with CAPN1, a cysteine protease involved in the influenza virus infection. Noticeably, Wu Y et al. found 10 lncRNAs differentially expressed in relation to exosomes through high-throughput sequencing of the whole blood of patients with recurrent COVID-19 and healthy people (47). It has been reported that exosomes in body fluids can be used as diagnostic markers for various diseases because they may reflect the pathological status of their derived cells, suggesting that identification and isolation of circulating lncRNAs in exosomes will contribute to the diagnosis of COVID-19.

### LncRNAs as Therapeutic Target

IFN is often used as the target of antiviral therapy, among which, IFN-α is a common interferon for antiviral therapy in COVID-19 patients with critical disease (106). As previously described, lncRNAs are emerging regulatory factors involved in numerous biological processes and have been predicted to play roles in the innate immune response through their associations with IFN-related pathways. For example, a decrease in MALAT1 expression induced by viral infection was found to promote IRF3 activation and type I IFN production, thereby playing an antiviral role in antiviral therapy (107). However, the discovery about the functions of lncRNAs is scarcely reported on the innate immune regulation related to interferon in COVID-19 patients. Up to now, the interactions between lncRNAs and IFN in SARS-CoV-2 are a freshly new frontier research area.

Of note, the immunity induced by viral infection can not only protect the human body, resist virus invasion, eliminate the virus and clear infection but also cause serious damage to the host (108). Thus, a well-coordinated immune response against viral infection is essential. In some patients infected with SARS-CoV-2, dysregulation of the cytokine response leads to a highly inflammatory state, suggesting that inhibition of the excessive inflammatory response may be an adjunct to the treatment of COVID-19 (66, 109). LncRNAs vary at the cell and tissue levels in different diseases and can trigger one or more biological processes by targeting several genes within the same biological network to maintain the stability of the intracellular environment. Currently, several studies have found that lncRNAs are abnormally expressed in COVID-19 and are involved in the occurrence of inflammation *via* the regulation of signaling pathways and transcriptional activation of multiple genes. Hence, lncRNAs can presumably target and bind to significant cytokine nucleotide sequences and have the potential to downregulate cytokine expression, which can ameliorate the proinflammatory immune response to COVID-19 infection, thereby mitigating cytokine storms in the process. Potential translational approaches to administering this technology in the clinical setting include antisense oligonucleotide knockdown, RNAi knockdown, and viral gene therapy (44). Studies have also elucidated the role of the TGF-β signaling pathway in SARS-CoV infection (103), and the hub lncRNAs and signaling pathways involved in the pathogenesis of SARS-CoV-2, including TGF-beta, can be considered potential therapeutic targets (102). TFs are the main regulators of dose-sensitive gene expression, and haploinsufficiency often leads to life-threatening illnesses. Moreover, many mechanisms have been developed to strictly regulate the expression and activity of TFs at the transcriptional, translational, and posttranslational levels. LncRNAs are spatially correlated with TFs across the genome. Swarr D T et al. reported that the lncRNA Falcor can fine-tune the expression of FoxA2, which maintains airway epithelial homeostasis and promotes regeneration (110). In the lungs, loss of the lncRNA Falcor can lead to chronic inflammatory changes and repair defects following airway epithelial injury. Accordingly, disruption of the Falcor-FoxA2 regulatory feedback loop leads to altered cell adhesion and migration, which in turn leads to chronic peribronchial airway inflammation and goblet cell metaplasia. Therefore, the full utilization of this regulatory feedback loop may play a certain role in inflammatory repair in patients with COVID-19.

In addition, based on the potential roles of IL-6 and the NLRP3 inflammasome in the immune response to SARS-CoV-2 infection, blocking these components may be a promising treatment strategy (111, 112). The data suggest that some ncRNAs exert anti-inflammatory effects, possibly by blocking IL-6 and inflammasome components, while others promote inflammation. Studies have shown that some anti-inflammatory drugs, including emodin, exert their biological actions by recruiting ncRNAs. Emodin inhibits lipopolysaccharide (LPS)-induced murine ATDC5 cell apoptosis and inflammation by upregulating the lncRNA taurine-upregulated gene 1 (Tug1), thereby blocking NF-κB signaling pathways and inflammatory cytokines, especially Il-6 (113). Additionally, multiple studies have found that MALAT1 and NEAT1 are differentially expressed in patients with COVID-19. Studies on the role of MALAT1 in inflammatory injury after lung transplantation have shown that silencing MALAT1 alleviates inflammatory injury by inhibiting neutrophil chemotaxis and immune cell infiltration at the infected site (94, 95). Therefore, the lncRNA MALAT1, as a factor regulating neutrophil chemotaxis, may be ubiquitous in severe cases, and its downregulation may play a role in alleviating inflammatory injury in COVID-19-positive patients. Recently, the monocyte immune response and viral replication induced by SARS-CoV-2 were found to require the support of aerobic glycolysis (114). The oxygen-sensing transcription factor HIF-1α can induce glycolysis, resulting in a proinflammatory state in SARS-CoV-2-infected monocytes (115). Previously, Chen F et al. found that the lncRNA HISLA in macrophages ensures the stability of the TF HIF-1α by inhibiting its binding to the PHD2 protein, thereby preventing its degradation and thus maintaining continuous activation of an aerobic state in tumor cells *via* HIF-1α signaling (116). This study demonstrated the potential of lncRNAs as signal transduction agents that can be transmitted between immune and tumor cells *via* extracellular vesicles (EVs) to promote aerobic glycolysis in cancer. Accordingly, in regard to HIF-1α playing a similar role in SARS-CoV-2 infection, the RNA interference-mediated silencing of HISLA may be a more feasible method to inhibit glycolysis in monocytes. Moreover, nucleic acid aptamers attached to the cell surface have been reported to target siRNAs delivery into bodily immune cells, including macrophages. In summary, the strategy of using aptamer-siRNA chimera-mediated HISLA knockdown specifically in associated macrophages for the treatment of COVID-19 needs to be further investigated.

Furthermore, treatments are urgently needed for some of the neurological symptoms caused by SARS-CoV-2. Several studies have found that some lncRNAs can promote the expression of antiviral polypeptides, thus inhibiting the proliferation of various neurotropic viruses. Polycomb repressive complex 2 (PRC2) is a protein complex with epigenetic regulatory activity that maintains histone modifications (117). EZH2, a catalytic component of PRC2, is a histone methyltransferase that can not only nonspecifically bind lncRNAs but also specifically bind new sites on lncRNAs to regulate their degradation (118, 119); EZH2 is highly expressed in many cancers (120). Recent studies have shown that a variety of neurotropic viruses can induce the expression of the lncRNA EDAL in neurons; this lncRNA can specifically bind to EZH2 and promote its degradation *via* the lysosomal pathway by regulating O-GlcAcylation at the T309 site to promote the expression of the antiviral polypeptide PCP4L1, ultimately leading to suppression of the proliferation of the viruses (121). Alternatively, patients with reported hyperinflammation caused by cytokine storms should be treated. As previously mentioned, the lncRNA DANCR was found to potentially regulate inflammatory cholinergic inhibition (46). Given that cholinergic signaling can affect both acute and long-lasting bodily and brain reactions, we hypothesize that lncRNAs contribute to the regulation of cholinergic signaling in response to COVID-19.

## Conclusions

In summary, lncRNAs are emerging as important players in the regulation of virus-mediated infection and the subsequent disease status. Along with advancements in research tools and techniques, numerous lncRNAs have been found to be differentially expressed in COVID-19 patients, and key lncRNAs for virus-host interactions have also been identified. On the one hand, the host uses lncRNAs to target and regulate the innate immune response to viral infections; on the other hand, viruses trigger the expression of molecules *via* the host and its own encoded lncRNAs to interfere with the host’s natural immune response and thereby create a suitable microenvironment for viral replication in cells. In addition, according to the characteristics of poorly conserved lncRNAs that are widely involved in cell proliferation, differentiation, apoptosis and other biological processes, the key targeted therapies for SARS-CoV-2-infected cells involve interfering with gene transcription, protein translation and extracore messengers. Although the functions and mechanisms of the evolutionarily conserved lncRNAs discovered thus far are potentially only the tip of the iceberg, this information provides new targets and ideas for the design of antiviral drugs. The present data clearly indicate the involvement of these lncRNAs in the progression of SARS-CoV-2 infection and pave the way for further exploring how lncRNAs are instrumental in regulating host-virus dynamics during RNA virus-mediated infections, particularly in HCoV-mediated diseases. However, the current research on lncRNAs has just started, still facing some of the following challenges, hence further research into their clinical application is needed. There are few reports on the role of lncRNA-RNA interaction network in viral infection, and the lack of research on clinical effectiveness greatly increases the challenge of clinical application of lncRNAs.

Several drugs are currently available for the clinical treatment of COVID-19, including potential antiviral drug candidates, monoclonal antibodies and glucocorticoids (122). Moreover, efforts are underway worldwide to develop a vaccine against SARS-CoV-2. By July 2021, 180 countries have initiated vaccination around the globe, with more than 3.3 billion doses administered. Despite the vaccine is effective in preventing infection, its side effects are inevitable. For example, the very rare occurrence of a mysterious blood-clotting disorder among some recipients of the Oxford–AstraZeneca COVID-19 vaccine (123), as shown in **Table 3**. Besides, recent reports suggest that CRISPR/Cas13-based gene editing is a potential alternative therapeutic strategy for viral infections, including SARS-CoV-2 (130). While promising, multiple checkpoints must be passed before these CRISPR-based strategies can be utilized in the clinic, such as proving that minimal off-target effects are exerted, designing effective *in vivo* drug delivery methods, and evaluating possible immunogenicity and cytotoxic effects, which could take several years. SARS-CoV-2 is reportedly mutating and evolving at a fairly rapid rate. In this case, RNA interference seems to be more appropriate, as it is relatively cost-effective and saves time and labor. The identification of lncRNAs that are altered or dysregulated during SARS-CoV-2 infection could be a new strategy for mitigating various symptoms in patients with COVID-19 and potentially prevent the progression of viral infection by limiting viral genome amplification and enhancing the immune response. Therefore, lncRNAs may prove to be helpful in designing RNA-based therapies against COVID-19 and become promising diagnostic biomarkers and therapeutic targets for COVID-19.

**Table 3:** Side effects in COVID-19 vaccine.

Vaccine	Platform	SARS-CoV-2 antigens	Side effects	Developer	Reference
ChAdOx1 nCov-19(AZD-1222)^a^	ChAd-vectored, non-replicating	Expressing S protein	Thrombotic thrombocytopenic	University of Oxford, AstraZeneca	(123)
Ad26.COV2-S^a^	Ad26-vectored, non-replicating	Expressing S protein	Thrombotic thrombocytopenic, cerebral venous sinus thrombosis	Johnson & Johnson	(124)
BNT162b1^a^	Lipid nanoparticle–mRNA	RBD of S protein	Anaphylactic reactions, Guillain-Barre Syndrome (GBS)	BioNTech, Pfizer, Fosun Pharma	(125–127)
v451	Protein subunit	Molecular clamp-stabilized S protein	False positive HIV tests	University of Queensland	(128)
mRNA-1273^a^	Lipid nanoparticle–mRNA	Expressing S protein	Anaphylactic reactions	Moderna, NIAID	(129)

^a^means that these vaccines selected for US Operation Warp Speed.

## Author Contributions

QY, FL and ML conceived the idea, analysis of literature, and writing of the manuscript. QY and YW collected and read the literature and revised the article. MZ and ML read through and corrected the manuscript. All authors contributed to the article and approved the submitted version.

## Funding

This work was supported by the National Natural Science Foundation of China [grant numbers 81800434], Grant of Sichuan Province Science and Technology Agency Grant [2019YJ0487], Foundation of Luzhou Municipal Science and Technology Bureau [2017LZXNYD-T05].

## Conflict of Interest

The authors declare that the research was conducted in the absence of any commercial or financial relationships that could be construed as a potential conflict of interest.

## Publisher’s Note

All claims expressed in this article are solely those of the authors and do not necessarily represent those of their affiliated organizations, or those of the publisher, the editors and the reviewers. Any product that may be evaluated in this article, or claim that may be made by its manufacturer, is not guaranteed or endorsed by the publisher.

## Glossary

**Table:**

COVID-19	coronavirus disease 2019
LncRNAs	long noncoding RNAs
ncRNAs	non-coding RNAs
IFN-I	type I interferons
SARS-CoV-2	severe acute respiratory syndrome coronavirus 2
TNF-α	tumor necrosis factor-α
MALAT1	metastasis-associated lung adenocarcinoma transcript 1
NEAT1	nuclear paraspeckle assembly transcript 1
DVT	deep vein thrombosis
EPCs	endothelial progenitor cells
CoVs	coronaviruses
ORF	open reading frame
S	spike
N	nucleocapsid
M	membrane
E	envelope
HE	hemagglutinin esterase
HCoVs	human coronaviruses
SARS-CoV	severe acute respiratory syndrome coronavirus
MERS-CoV	Middle East respiratory syndrome coronavirus
ACE2	angiotensin-converting enzyme 2
DPP4	dipeptidyl peptidase 4
NTD	N-terminal domain
CTD	C-terminal domain
RBDs	RNA-binding domains
TMPRSS2	transmembrane serine protease 2
TLR7	toll-like receptor 7
TFs	transcription factors
NF-κB	nuclear factor kappa B
IRF7	interferon regulatory factor 7
ARDS	acute respiratory distress syndrome
MOF	multiple organ failure
NRP1	neuropilin-1
ICSR	inflammatory cytokine storm response
RPL6	ribosomal protein
KDELR3	endoplasmic reticulum protein retention receptor 3
TubA1A	tubulinα1
BALF	bronchoalveolar lavage fluid
PBMC	peripheral blood mononuclear cells
RIG-I	retinoic acid-induced gene I
HLA-II	human leukocyte antigen class II
HIV	human immunodeficiency virus
IL-6	interleukin-6
SFPQ	splicing factor proline-and glutamine-rich
NLRP3	Nod-like receptor family pyrin domain-containing 3
MDSCs	myeloid-derived suppressor cells
ceRNAs	competitive endogenous RNAs
LATs	latency-associated transcripts
PRV	pseudorabies virus
IFN-α	interferon-α
ISGs	IFN-stimulating genes
HCV	hepatitis C virus
IFI6	interferon-inducible protein 6
IAV	influenza A virus
NBHE	normal bronchial human epithelial
TF	transfer factor
LPS	lipopolysaccharide
Tug1	taurine-upregulated gene 1
EVs	extracellular vesicles
HIF-1α	hypoxia-inducible factor-1α
PRC2	polycomb repressive complex 2
EZH2	enhancer of zeste homolog 2
CRISPR	clustered regularly interspaced short palindromic repeats
